# A Treatise on Ruptures

**Published:** 1843-06

**Authors:** 


ART. VIII.—*A Treatise on Ruptures.* By W. LAWRENCE, F. R. S., Surgeon extraordinary to the Queen, &c. From the fifth London edition. Revised, corrected and enlarged. pp. 480, 8vo. Lea and Blanchard; Philadelphia, 1843.

This work is no less excellent than the former by the same author, and, like it, has long since taken rank as one of the very highest authority. Both of them indeed bear the impress of the accomplished and well-stored mind from which they emanate. There is a completeness and a finish about the productions of Mr. Lawrence (conspicuous in both these works) rarely to be seen in those of any other writer. His extent of learning and research—all of ancient and modern medical literature being thoroughly searched for materials—is unequalled; and all thus collected, is compact in that singularly felicitous style, for which he is remarkable. He gives you all that is known on a subject—all that has been said or written about it, not crudely and *undigestedly*, but after having well weighed its merits, his opinion of which he does not hesitate to express. These are the labors that possess true value, and that must endure.

The corrections, revisions and additions, mentioned in the title, were made by the author to the fifth edition published in London. C.

